# Hippocampal Gene Expression of Deiodinases 2 and 3 and Effects of 3,5-Diiodo-L-Thyronine T2 in Mouse Depression Paradigms

**DOI:** 10.1155/2013/565218

**Published:** 2013-12-10

**Authors:** Natalyia Markova, Anton Chernopiatko, Careen A. Schroeter, Dmitry Malin, Aslan Kubatiev, Sergey Bachurin, João Costa-Nunes, Harry M. W. Steinbusch, Tatyana Strekalova

**Affiliations:** ^1^Institute of Physiologically Active Compounds, Russian Academy of Sciences, Severnii proesd 1, Chernogolovka, Moscow Region 142432, Russia; ^2^Timantti AB, Sundbyberg 104, 174 07 Stockholm, Sweden; ^3^Department of Preventive Medicine, Maastricht Medical Center in Annadal, Becanusstraat 17 A0, 6216 BX Maastricht, The Netherlands; ^4^Carbone Cancer Center, University of Wisconsin, WIMR 3016, 1111 Highland Avenue, Madison, WI 53705, USA; ^5^Institute of General Pathology and Pathophysiology, Russian Academy of Medical Sciences, Baltiyskaia 8, Moscow 125315, Russia; ^6^Department of Neuroscience, School for Mental Health and Neuroscience, Maastricht University, Universiteitssingel 40, NL 6229 ER Maastricht, The Netherlands; ^7^Institute for Hygiene and Tropical Medicine, New University of Lisbon, Rua da Junqueira 96, 1349-008 Lisbon, Portugal

## Abstract

Central thyroid hormone signaling is important in brain function/dysfunction, including affective disorders and depression. In contrast to 3,3′,5-triiodo-L-thyronine (T3), the role of 3,5-diiodo-L-thyronine (T2), which until recently was considered an inactive metabolite of T3, has not been studied in these pathologies. However, both T3 and T2 stimulate mitochondrial respiration, a factor counteracting the pathogenesis of depressive disorder, but the cellular origins in the CNS, mechanisms, and kinetics of the cellular action for these two hormones are distinct and independent of each other. Here, Illumina and RT PCR assays showed that hippocampal gene expression of deiodinases 2 and 3, enzymes involved in thyroid hormone regulation, is increased in resilience to stress-induced depressive syndrome and after antidepressant treatment in mice that might suggest elevated T2 and T3 turnover in these phenotypes. In a separate experiment, bolus administration of T2 at the doses 750 and 1500 mcg/kg but not 250 mcg/kg in naive mice reduced immobility in a two-day tail suspension test in various settings without changing locomotion or anxiety. This demonstrates an antidepressant-like effect of T2 that could be exploited clinically. In a wider context, the current study suggests important central functions of T2, whose biological role only lately is becoming to be elucidated.

## 1. Introduction

Despite advances in the pharmacotherapy of depression, many patients fail to respond to standard antidepressants. This requires new treatment and augmentation approaches to be developed. Further elaboration of a potential of the brain thyroid system to be targeted to elicit an antidepressant action can be one of the most promising strategies. Central thyroid hormone synthesis was demonstrated in the dentate gyrus of the hippocampus, the septum, amygdala, and the olfactory bulb [1–5]. The action of thyroid hormones in the CNS is considered to be independent of peripheral thyroid hormones [5–7]. At very low doses, brain thyroid hormones induce profound effects on the CNS. For example, they enhance hippocampal neurogenesis [8] and the secretion of neurotrophins, including BDNF, either directly or via monoamine receptors, and they activate PI3 K-Akt signaling through integrin receptors [9–11]. Deficiency of brain thyroid hormone production contributes to reduced central serotonin activity and development of depression [6, 12].

A number of studies suggest that both 3,3,5-triiodo-L-thyronine (T3) and 3,5,3′,5′-tetraiodo-L-thyronine (T4) thyroid hormones could be promising adjunct therapy in patients refractory to tricyclics and selective serotonin reuptake inhibitors (SSRI) [13, 14]. Hitherto, it was not clear whether 3,5-diiodo-L-thyronine (T2), which has been recently identified as functionally active metabolite of T3 in periphery in *in vivo* and *in vitro *systems, might have similar effects to T3 that might be exploited in a clinic. New evidence does suggest that T2 may mediate the effects of antidepressant therapy in the brain. A two-week intraperitoneal administration of the tricyclic antidepressant desipramine in the rat induces the expression of T2 in the amygdala [2]. This was paralleled by increased concentrations of T3 in nuclei but in contrast to T2 expression not in the mitochondria. These desipramine-induced changes in T2 were accompanied by an increase in the concentrations of succinate dehydrogenase in the mitochondria, suggesting their elevated functional activity [2]. Other experiments of the same group [1] have shown the presence of metabolites of T2 in several brain areas including the hippocampal formation and septum that points to the occurrence of T2 itself in the limbic system. Extensive study of Eravci and coworkers [15] has shown significant changes in the hippocampal formation and amygdala in the key enzymes regulating the levels of thyroid hormones, deiodinases 2 (DIO2) and 3 (DIO3) of the rats which were dosed with antidepressant and antipsychotic agents or subjected to a 24 h sleep deprivation, a commonly used nonpharmacological antidepressant treatment. Thus changes in the levels of thyroid hormones by their synthesis or degradation in the limbic system generally and in the hippocampus in particular are implicated in depressive traits and antidepressant response.

T2 was recently found to increase resting metabolic rate and prevent diet-induced insulin resistance through the stimulation of the mitochondrial respiratory chain; the latter mechanism is in itself an emerging target of new antidepressant therapies [16–21]. These and other studies have revealed intriguing cellular effects of T2 that seem to be distinct from that of T3. Unlike T3, T2 enhances mitochondrial respiration by a nuclear-independent mechanism and neither does act via thyroid hormone receptor beta, nor via AMP-activated protein kinase. T2 was demonstrated to activate reactions involved in substrate oxidation, affecting both cytochrome c reducers and cytochrome c oxidizers [20, 21], and besides it effects on respiratory chain, it was suggested to increase the downstream mechanisms that are involved in mitochondrial biogenesis [17]; these and other effects of T2 on mitochondrial pathways were shown to be distinct from the effects of T3 [22]. In contrast to the lasting l effects of T3 on metabolic rate and mitochondrial respiration, with onset delayed for 48 h, the action of T2 appears within the first few hours and does not persist for longer than 48 h and is not sensitive to actinomycin D [5, 20, 22]. T2 was found to evoke selective thyromimetic activity *in vitro* and *in vivo* that is divergent from that of T3 [23]. This and other mounting evidence, suggest that the effects of T2 do not merely mimic those of T3 but, instead, involve distinct mechanisms that seem likely to be related to stimulatory action on mitochondria.

Intriguingly, there is growing evidence to suggest that antidepressants can stimulate the mitochondrial respiratory chain directly and indirectly and suggest that these effects are implicated in the stress response and the pathogenesis of a depressive-like state. For instance, thiazolidinediones, which act as potent sensitizers of the neuronal insulin receptor, enhance brain glucose utilization though increased neuronal mitochondrial biogenesis [24], decrease neuronal damage [25], induce an antidepressant-like effect in the tail suspension and forced swim tests in mice [26], and show clinical efficacy in patients with major depression [27, 28]. Our recent studies on mice have indicated that dicholine succinate, a molecule that stimulates insulin-dependent H_2_O_2_ production of the mitochondrial respiratory chain, decreased signs of stressed-induced anhedonia in a sucrose test, immobility in the forced swim model, and hippocampal gene expression [29].

So far, limited efforts have been made to address potential central functions of T2 using animal models of depression. The hippocampus, as part of the limbic system, was chosen as a focus of the present study based on the striking differences between resilient versus susceptible to stress-induced depressive-like state mice in the mitochondrial gene expression of this structure [30, 31]. Using the above-mentioned paradigm of stress-induced anhedonia in C57BL6J mice, we addressed whether hippocampal expression of the key enzymes regulating the levels of thyroid hormones, DIO2 and DIO3, is altered in the hippocampus of animals with depressive-like features. Separately, we also assessed the effects of a bolus administration of 3,5-diiodo-L-thyronine (T2) in mice in the tail suspension test, a common test of depressive-like behaviour, and in supplementary paradigms for anxiety and locomotion, such as dark/light and novel cage tests. The efficacy of the treatment with T2 was evaluated on two laboratory strains, C57BL6J and CD1 mice. A selection of a two-day tail suspension paradigm as a test for a depressive-like behavior [32] and of single dosing were based on previously reported studies, in which T2 induced marked effects in rats within the first hours after administration [16, 33] and chronic administration of T2 evoked thyromimetic action in rats [23] that we wished to avoid in our study. Therefore, T2 was applied to mice intragastrically as a bolus injection 30 min prior to or 2 h after the first test session.

## 2. Materials and Methods

### 2.1. Animals and Housing

Male C57BL6J and CD1 mice were 3-month old. Two–five-month-old Wistar rats of the same age were used for predator stress. Mice were obtained from Charles River (Janvier, l'Arbresle Cedex, France) and rats from the Medical Faculty of New University of Lisbon, Lisbon, Portugal. 14 days before the behavioural experiments, mice were single housed under a reverse 12 h : 12 h light-dark cycle (lights on: 21:00 h) in standard laboratory conditions (22 ± 1°C, 55% humidity, food, and water *ad libitum). *All experiments were carried out in accordance with the European Committees Council Directives and had been approved by the state governmental bodies of animal care and welfare.

### 2.2. Chronic Stress Experiment 

#### 2.2.1. Experimental Conditions and Study Outline

**Figure 1:**
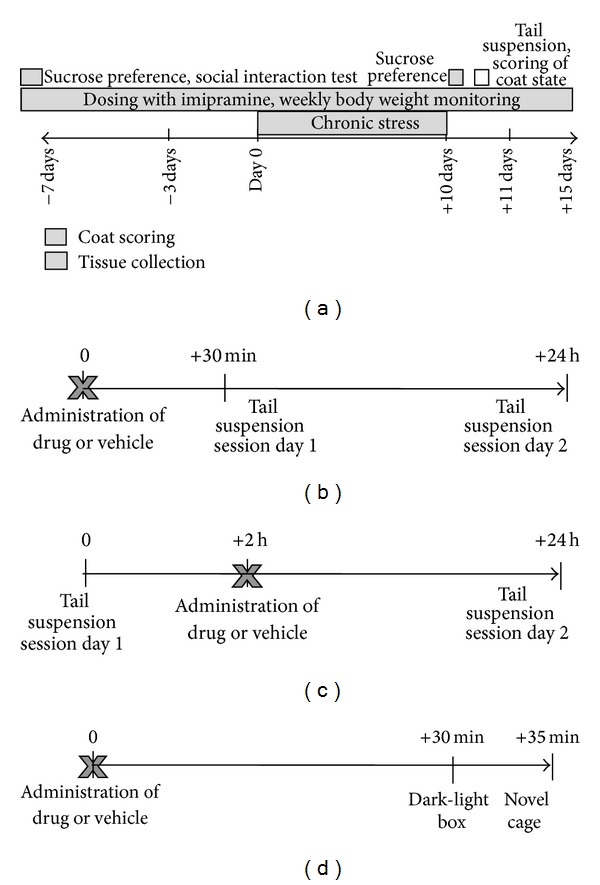
Schematic timeline of the chronic stress (a) and tail suspension ((b)–(d)) experiments.

Parameters of social behaviour were determined one week before the chronic stress procedure in a social interaction test as described elsewhere [30, 34, 35]. Body weight and baseline preference for a 1% sucrose solution (see *Sucrose Test*) were recorded (Figure 1(a)). The experimental and control groups were balanced upon these parameters. Together, 40 mice were assigned to a stress group and 24 controls constituted a nonstressed control group. Among animals from a stress group, twenty mice received no treatment and twenty were treated with imipramine. Control mice were neither treated (*n* = 14) nor treated with imipramine (*n* = 10). In control and stress groups, imipramine (7 mg/kg/day) was administrated via drinking water starting 7 days prior to the onset of stress and lasting the entire duration of the stress procedure. The current antidepressant treatment was applied as described elsewhere [29].

Additionally to baseline measurements, a sucrose consumption test was performed on the 10th day of the chronic stress procedure. On the next day, mice were tested in the tail suspension test and scored for a coat disintegration, and their body weight was evaluated. Five days after the termination of the stress procedure, mice were sacrificed for gene expression analysis (Figure 1(a)). 

*(1) Chronic Stress Procedure*. This study uses a recently validated variant of a 10-day stress protocol [29, 34, 35] comprising night time rat exposure and day time application of two stressors: a social defeat and restraint stress. Between the hours of 10:00 and 17:00, social defeat for 30 minutes and restraint stress were employed for 2 hours with an inter-session interval of at least 4 hours.

*Rat Exposure While in a Small Container*. Mice were introduced to transparent glass cylindrical containers (15 cm × 0.8 cm) and placed into the rat cage (15 h exposures were performed between 18.00 and 09.00).

*Social Defeat Stress*. Social defeat procedures took place during the dark phase; to enable a visual control over the resident-intruder confrontation, the test was carried out under red light. In a preliminary test, aggressive individuals of the CD1 mouse strain that were able to attack the counter partners in less than 60 sec without injuring them were selected for this procedure; these animals were introduced in the home cages of mice from the stress group during social defeat sessions. Social interaction was set up in the home cage of stressed animals as it enhances the impact of the stress procedure in a lasting manner. In a variant procedure, a defeated animal is left in chronic contact with the olfactory cues of the aggressive intruder, such that exposure to a psychological stressor is chronic, although the actual agonistic experience is intermittent. Average duration of each session was 30 min in accordance with commonly used protocols. During social defeat stress, test mice typically showed flight response, submissive posture, and vocalization. Pairs of animals were carefully observed in order to exclude any physical harm. In rare cases of its incidence, aggressive individuals were immediately removed from the cage of resident mice.

*Restraint Stress*. Animals were placed inside plastic tubes (internal diameter is app. 26 mm) during the dark phase of the light cycle. Small standard pieces of tissue paper were inserted in the tubes to restrict animals' activity.

*(2) Assessment of Stress Effects*

*Sucrose Test*. Animals were given 8 hours of free choice between two bottles of either 1% sucrose or normal drinking water. At the beginning and end of the period, the bottles were weighed and consumption was calculated. The beginning of the test started with the onset of the dark (active) phase of animals' cycle. To prevent the possible effects of side preference in drinking behaviour, the position of the bottles in the cage was switched at 4 hours, halfway through testing. No previous food or water deprivation was applied before the test. Other conditions of the test were applied as described elsewhere [24]. The 1% sucrose solution is used in tests performed during baseline and chronic stress application. Percentage preference for sucrose is calculated using the following formula:

(1) sucrose  preference =[V(Sucrose  solution)V(Sucrose  solution)+V(Water)]×100%.

A decrease in sucrose preference to a level below 65% measured at the 10th day of continuous stress application was taken as a criterion for anhedonia. This criterion was based on the fact that none of the control animals exhibited <65% preference for sucrose at that time point of the study and, accordingly, mice exhibiting a sucrose preference of <65% were defined as susceptible to stress-induced anhedonia. Mice that had undergone stress but maintained a sucrose preference of >65% were defined as resilient to this state. In addition, our previous results indicated that mice matching this criterion showed a depressive-like syndrome [35]. This procedure induces anhedonia in a considerably shorter time than previously validated models by increasing the daytime stress load.

*Tail Suspension Test*. The protocol used in this study was adapted from a previously proposed procedure [36]. Mice were subjected to the modified tail suspension by being hung by their tails with adhesive tape to a rod 50 cm above the floor for 2 min. Two animals were tested simultaneously in a dark room where only the area of the modified tail suspension construction was illuminated by a spotlight from the ceiling; the lighting intensity on the height of the mouse position was 25 Lux. The trials were recorded by a video camera positioned directly in front of the mice, while the experimenter observed the session from a distance in a dark area of the experimental room. This procedure was carried out twice with a 24 h interval between tests, similarly to previously reported protocols [32]. The latency of the first episode of immobility and the total duration of this behaviour were scored manually according to the protocol that was previously validated with automated scoring using CleverSys software [36]. In accordance with the commonly accepted criteria of immobility, the immobility behaviour was defined as the absence of any movements of the animals' head and body. The latency of immobility was determined as the time between the onset of the test and the first bout of immobility.

*Coat State Scoring*. A coat state was scored in a blinded fashion to assess a stress-related disintegration of fur. Scores from 1 (very poor) to 5 (excellent) were assigned to each animal by two independent experimenters; the average note was taken. This parameter was earlier shown to correlate with a development of stress-induced anhedonia as it reflects self-grooming behaviour that is dependent on hedonic sensitivity [36].

*Brain Dissection, RNA Isolation, and Illumina Microarray Gene Expression Profiling*. Mice were sacrificed by cervical dislocation. RNA extraction was performed from microdissected snap-frozen hippocampi using RNeasy RNA extraction kit with DNaseI treatment, as previously described (Qiagen, Hilden, Germany; [34]).

Gene expression profiling was performed using Illumina technology (Integragen, Evry, France, and Northwestern Chicago University, USA) with the hippocampi of mice from nonstressed control (drug-naive or treated with imipramine), stressed resilient, and anhedonic groups and from stressed imipramine-treated mice (five animals per each group were analyzed). Total RNA samples were hybridized to Illumina BeadChips (MouseRef-8 v2 Expression BeadChip; Illumina, Inc. San Diego, CA, USA) which were prepared using the Illumina TotalPrep RNA Amplification kit (Applied Biosystems/Ambion, Carlsbad, CA, USA); the samples were assigned to the chips in random order with the constraint that no two samples from the same group were assigned to the same chip to avoid confounding of experimental groups with the chips. Microarray data were analyzed using standard analysis procedures, which included assessment of the overall quality of array data and statistical evaluation of differentially expressed genes (IntegraGen, Evry, France). Once the quality of array data was confirmed, the Gene Chip Operating System (Illumina, Inc., San Diego, CA, USA) was used to calculate signal intensities, detection calls, and their associated *P* values for each transcript on the array. Gene expression was normalized to the expression of the housekeeping gene, beta-actin, due to its stable expression, and calculated as percent mean of the respective control group (pharmacologically naive or treated with imipramine). Differences in gene expression between groups were evaluated using unpaired two-tailed *t*-test.

*Real-Time PCR (RT-PCR)*. 1 *μ*g total RNA was converted into cDNA as described elsewhere [34]. RT-PCR was run using SYBR green based technology (Primer Design Ltd., Southampton, UK). Standard curves were generated using previously generated samples to enable normalization to the housekeeping gene glyceraldehyde-3-phosphate dehydrogenase (GAPDH; forward primer ACCCCTTCATTGACCTCAACTACATG; reverse primer CCTTCTCCATGGTGGTGAAGAC) using the Pfaffl method as described elsewhere [37]. Expression of DIO2 was assessed using a forward primer GATGCTCCCAATTCCAGTGT and reversed primer TGAACCAAAGTTGACCACCA; expression of DIO3 was assessed using a forward primer CCGCATATGGTGCCTATTTT and reversed primer GCCCACCAATTCAGTCACTT. All samples were run in duplicate. Cycling was performed at 95°C for 5 min followed by a 45 cycle amplification at 95°C for 10 s, then at the annealing temperature for 15 s and at the temperature 72°C for 20 s. Results of the qPCR measurements were expressed as Ct values, where Ct is defined as the threshold cycle of PCR at which amplified product was 0.05% of normalized maximal signal. We used the comparative Ct method and computed the difference between the expression of the gene of interest and GAPDH expression in each cDNA sample (2-ΔΔ Ct method). Results are expressed as relative-fold change compared to control animals.

### 2.3. Tail Suspension Experiment

On the basis of gene expression data, the further aim of our study is to examine the effects of bolus T2 administration on the immobilization behaviour in the tail suspension test in C57BL6J and CD1 mice. Supplementary tests for anxiety-like behaviour and locomotion were carried out on the same mouse strains in order to rule out potential confounds with the evaluation of behavioural despair after the treatment with T2.

First, we investigated the effects of a bolus oral gavage of T2 (see *drug administration *below) to C57BL6J mice at the dose of 250 mcg/kg either 30 min before (*n* = 7) or 2 h after (*n* = 8) the first tail suspension session of the test (Figures 1(b) and 1(c)), as described previously [26, 32, 36]; control groups constituted 8 mice in each experiment. Second, T2 was used at the dose of 750 mcg/kg prior to the tail suspension session (*n* = 8) or 2 hrs thereafter (*n* = 8); control groups constituted 8 mice. Finally, a dose of 1500 mcg/kg was applied 30 min prior to the first tail suspension session in CD1 mice (*n* = 10); control group was formed by 10 mice. The same vehicle was injected into controls. A selection of doses of T2 and the timing of dosing were based on previously reported studies, in which it induced marked effects in rats within the first hours after administration [16, 33].

In an additional study, we addressed the question of whether the administration of 750 mcg/kg of T2 in C57Bl6J and 750 mcg/kg of T2 or 1500 mcg/kg of this drug in CD1 mice interferes with anxiety and locomotion in a dark/light and novel cage tasks in previously calibrated protocols [36, 38, 39]. Dark/light and novel cage tasks were applied with a 5 min interval 30 min after a dosing (Figure 1(d)). All groups constituted 9 mice.

#### 2.3.1. Dark/Light Box

The dark/light box (TechnoSmArt, Rome, Italy) consisted of two plexiglass compartments, one black/dark (15 cm × 20 cm × 25 cm) and one lit (30 cm × 20 cm × 25 cm), connected by a tunnel. Anxiety-like behaviour was assessed by earlier validated measures. Mice were placed into the dark compartment, from where they could visit the lit box (light intensity 25 Lux). The latency of the first exit to the light compartment, the total duration of time spent in the lit box, and the number of visits to this anxiety-related compartment were scored by visual observation over 5 min.

#### 2.3.2. Novel Cage Test

The novel cage test was performed to assess vertical activity [35, 36, 39]. Mice were introduced into a standard plastic cage; the size of their home cage filled with small amounts of fresh sawdust. The number of exploratory rearings was counted under red light during a 5 min period. The testing was carried out in a dark quiet room in morning hours. Behaviour was videotaped and analyzed by trained observers blind to the animals' treatment.

#### 2.3.3. Drug Administration

Imipramine (Sigma-Aldrich, St. Louis, MO, US) was dissolved in tap water; the solution was freshly prepared every 2-3 days and placed in lightproof drinking bottles. The calculation of the concentration of imipramine in drinking water was based on the previously evaluated mean volume of daily water consumption in C57BL6 mice that was about 3 mL and on the dosage of treatment. Dosage for imipramine was set at 7 mg/kg/day as previous studies showed that chronic administration of imipramine at 15 mg/kg/day with drinking water, but not 7 mg/kg/day, significantly affects sucrose intake and locomotor behaviour in naive C57BL6N mice [39]. Imipramine was delivered with drinking water starting 1 week before the onset of stress and then throughout the entire duration of the chronic stress procedure.

3,5-Diiodo-L-thyronine T2 (Sigma-Aldrich, Cambridge, MA, USA) was first dissolved in 100% DMSO (Sigma-Aldrich, St. Louis, MO, US), the solution was diluted, so the final concentration of DMSO that was 0.2% and administrated to mice via a single intragastrical administration. The same treatment method was applied to administer a vehicle 0.2% DMSO solution. A separate control group of mice was injected with saline; no effects of 0.2% DMSO solution were found in protocols applied here of the tail suspension tests (Strekalova and Chernopiatko, *unpublished results*). The volume of T2 and vehicle injections was 0.1 mL/10 g of body weight.

### 2.4. Statistics

Data were analysed with GraphPad Prism version 5.00 for Windows, San Diego, CA. One- and two-way ANOVA were used followed by a Mann-Whitney *U*-tests posthoc comparisons. Two-tailed unpaired *t*-tests were applied for two-group, two-tailed comparisons of independent data sets with normal distribution. The level of confidence was set at 95% (*P* < 0.05) and data are shown as mean ± SEM.

## 3. Results

### 3.1. Assessment of Anhedonia Induction

In the model employed, the induction of a depressive-like state by stress exposure was defined by the occurrence of behavioural manifestations of anhedonia, as determined by a decrease in preference for a 1% sucrose solution to below 65% [35]. Previous studies have shown that the occurrence of stress-induced anhedonia is associated with a number of behavioural, physiological and molecular features of a depressive state [29–31, 34–36].

**Figure 2:**
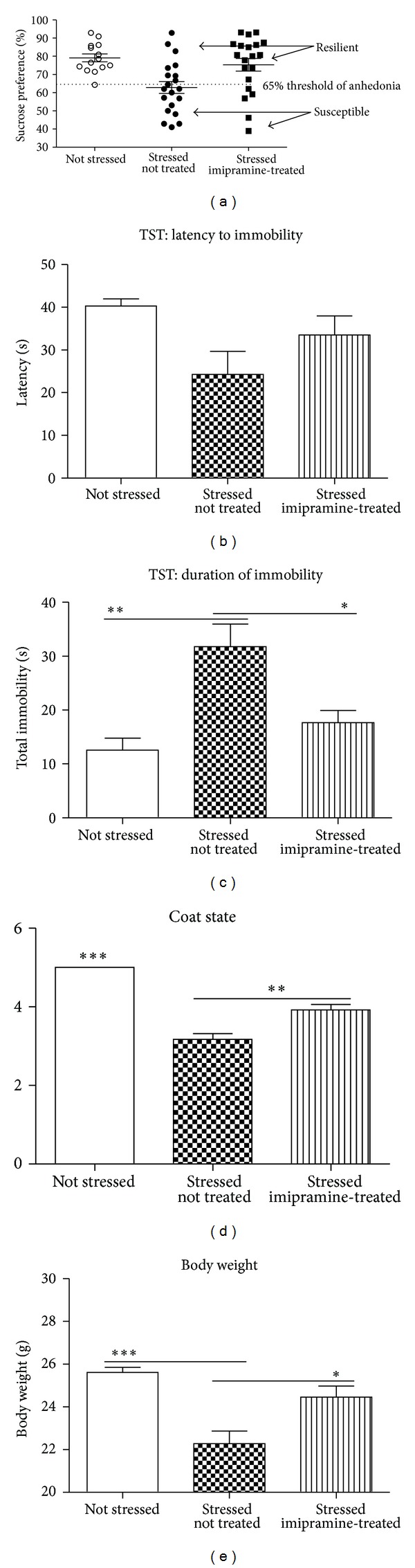
Chronic stress induces physiological and behavioural changes in a subgroup of anhedonic mice, which are not evident in mice resilient to stress. (a) A loss of sucrose preference was used to define the susceptible group, which was measured after the termination of a 10-day stress paradigm. (b) Latency to immobility in the tail suspension test was significantly shorter in stressed drug-naive animals than in control and stressed imipramine-treated groups. (c) The duration of immobility in the tail suspension test was significantly longer in stressed drug-naive than in control and stressed imipramine-treated groups. (d) In comparison to control mice, the score of coat state was significantly decreased in stressed nontreated mice and, to lesser extent, in stressed imipramine-treated animals. (e) Body weight was significantly reduced in both stressed group; it was significantly higher in imipramine-treated than in nontreated stressed groups. Data are mean ± SEM; *P < 0.05, **P < 0.01, and ***P < 0.001; TST: tail suspension test.

When assigned to experimental groups, but before being subjected to the chronic stress procedure, groups did not differ in terms of sucrose preference, intake of sucrose solution and water, or body weight (*P* > 0.05; one-way ANOVA; *data not shown*). Two-way ANOVA revealed a significant effect of stress on sucrose preference (*F*_1,59_ = 7.671; *P* = 0.0075). A *Post hoc *Mann-Whitney test showed a significant difference in sucrose preference between control and the nontreated stressed group (*U* = 47.00; *P* = 0.0012), but when treated with imipramine there was no difference from control and stressed groups in terms of sucrose preference (*U* = 135.0; *P* = 0.8748). This suggests that imipramine prevents the reduction in sucrose preference for the stressed cohort (Figure 2(a)). 60% of nontreated stressed mice (12 out of 20) and 25% of imipramine-treated stressed animals (5 out of 20) showed a sucrose preference below 65% and were defined as anhedonic (susceptible).

### 3.2. Effects of Treatment on Immobility Behaviour

Previous studies have shown that stress-induced anhedonia, measured by the sucrose preference test, is frequently associated with increased “behavioural despair” in the Porsolt forced swim and tail suspension paradigms [40]. Onset of immobility (behavioural despair) in the latter model is defined as the point at which the animal stops struggling for longer than 1 second. Latency to immobility was significantly altered by stress as revealed by two-way ANOVA (*F*_1,49_ = 17.70; *P* = 0.0001). *Post hoc *Mann-Whitney tests showed a strong trend towards a significant reduction in latency to immobility for stressed nontreated animals compared with control mice (*U* = 60.50; *P* = 0.0889); there was no significant difference between stressed and control mice that received imipramine (*U* = 76.50; *P* = 0.2213; Figure 2(b)). Time spent immobile showed a significant main effect of stress (*F*_1,49_ = 24.79; *P* < 0.0001); stressed mice were immobile for significant longer than controls (Figure 2(c)). Posthoc analysis showed significant differences between control and stressed nontreated groups (*U* = 27.00; *P* = 0.0012) but not between control and stressed imipramine-treated group (*U* = 67.50; *P* = 0.1056) suggesting that applied treatment prevents the behavioural despair induced by the chronic stress paradigm.

### 3.3. Evaluation of Coat Scores

Before the onset of the chronic stress procedure, all mice had good coat quality, with no significant difference between the groups (*data not shown*). After completion of the chronic stress procedure, a two-way ANOVA comparison showed significant main effects of both stress (*F*_1,60_ = 125.8; *P* < 0.0001); and treatment (*F*_1,60_ = 8.416; *P* = 0.0052; Figure 2(d)). All stressed mice showed significantly lower scores of coat state than control-treated animals (*P* < 0.001). However, imipramine-treated animals exhibited higher scores of coat state compared with the nontreated group (*U* = 80.00; *P* = 0.0009).

### 3.4. Changes in Body Weight

At the end of the chronic stress paradigm, there was a significant difference in body weight between control and stressed animals (two-way ANOVA; *F*_1,60_ = 65.39; *P* < 0.0001), and stressed drug-naive group showed a significant decrease in body weight compared with controls (*U* = 21.5, *P* < 0.0001 for stressed nontreated animals), while imipramine-treated mice did not show such a change (*U* = 100.5; *P* = 0.1718; Figure 2(e)). Stressed mice treated with an antidepressant were significantly heavier than those that were not treated pharmacologically (*U* = 111.5; *P* = 0.0172; Figure 2(e)).

### 3.5. Expression of Genes Encoding Deiodinases 2 and 3 in the Hippocampus of Stressed Mice

**Figure 3:**
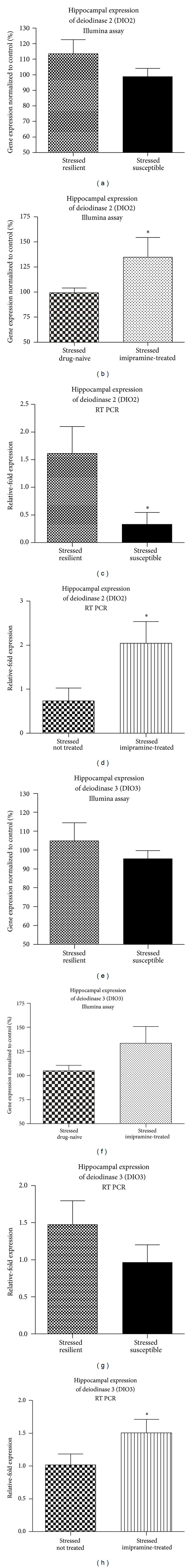
Hippocampal gene expression of deiodinases 2 and 3 in a chronic stress depression model. Illumina assay has pointed to (a) a nonsignificant decrease in DIO2 expression in susceptible versus resilient mice and (b) significant elevation of DIO2 expression in stressed imipramine-treated group in comparison with stressed drug-naive animals. RTPCR analysis showed a significant (c) decrease in DIO2 expression in susceptible versus resilient mice and (d) increase in DIO2 expression in stressed imipramine-treated group in comparison with stressed nontreated mice. As for DIO3 expression, Illumina assay has revealed no significant differences in its expression between (e) susceptible versus resilient mice and (f) stressed imipramine-treated stressed drug-naive groups. RTPCR analysis demonstrated a lack of significant differences with the former comparison (g), but (h) showed a significant increase in DIO3 expression in stressed imipramine-treated group in comparison with stressed nontreated mice *P < 0.05.

There was a trend towards elevated DIO2 expression for resilient versus susceptible mice as shown by Illumina microarray analysis (*P* = 0.10; *t* = 1.362, df = 6; unpaired *t*-test; Figure 3(a)), and RTPCR revealed an increase in DIO2 expression in resilient versus susceptible mice (*U* = 2.000; *P* = 0.0488; Mann-Whitney test; Figure 3(c)). Both Illumina profiling and RTPCR assays have shown that DIO2 was significantly augmented in the stressed imipramine-treated group in comparison with stressed drug-naive animals (*P* = 0.04; *t* = 1.797; df = 16; *t*-test and *U* = 3.000; *P* = 0.0424; Mann-Whitney test, resp.; Figures 3(b) and 3(d)). There was a trend toward elevated DIO3 expression for resilient versus anhedonic mice as shown by Illumina assay (*P* = 0.11; *t* = 0.9755; df = 7; *t*-test; Figure 3(e)) and RTPCR analysis (*U* = 3.000, *P* = 0.1250; Mann-Whitney; Figure 3(g)). Illumina microarray has demonstrated a nonsignificant increase in the DIO3 expression in imipramine-treated animals over this measure in stressed drug-naive group (*P* = 0.137; *t* = 1.557; df = 18; *t*-test; Figure 3(f)). Following this trend, RTPCR revealed significantly elevated level of DIO3 expression in the stressed imipramine-treated group in comparison with stressed drug-naive animals (*U* = 3.000; *P* = 0.0420; Mann-Whitney test, resp., Figure 3(h)). Of note, in control nonstressed mice, the expression did not differ for either gene between drug-naive and imipramine-treated groups (*data not shown*).

### 3.6. 3,5-Diiodo-L-Thyronine T2 Reduces Immobility Time in the Tail Suspension Test

**Figure 4:**
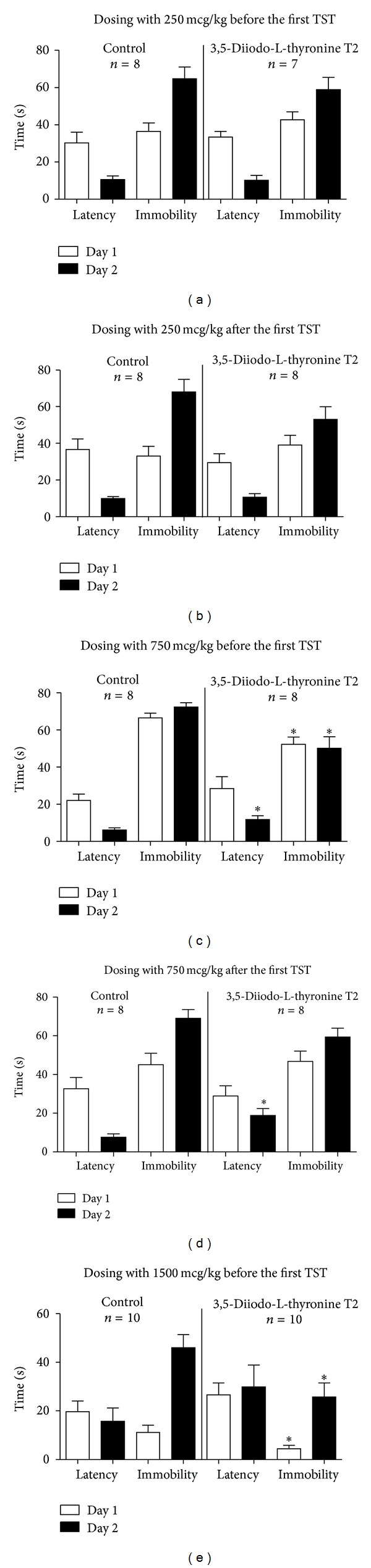
3,5-diiodo-L-thyronine T2 reduces immobility time in the tail suspension test. On Day 1 and 2 of the tail suspension test, mice treated with 250 mcg/kg of T2 30 min before (a) or 2 h after (b) the first session of testing showed no differences in the latency of immobility and in the duration of this behaviour. (c) Mice treated with 750 mcg/kg of T2 30 min prior to the first test session had a trend towards longer latency of immobility on Day 1 and significant increase in this parameter on Day 2; the duration of immobility was significantly reduced. (d) Animals that received the dose of 750 mcg/kg of T2 2 h after the first session of testing had significant increase in the latency of immobility on Day 2, and the duration of immobility had a tendency to a decrease. On Day 1, prior to treatment, mice from two groups had similar behavioural scores. (e) Mice dosed with 1500 mcg/kg of T2 30 min prior to the first test session had a trend towards longer latency of immobility on Days 1 and 2; the duration of immobility was significantly reduced. *P < 0.05 versus control.

The parameters of immobility in mice dosed with 250 mcg/kg of T2 in either protocol of the tail suspension test were unaltered (Figures 4(a) and 4(b)). C57BL6J and CD1 mice dosed prior to the first testing with 750 mcg/kg or 1500 mcg/kg, respectively, had significantly reduced immobility on Day 1 (*P* = 0.008, *t* = 3.076, df = 14 and *P* = 0.025, *t* = 2.089, and df = 18) and Day 2 (*P* = 0.0205, *t* = 3.327, df = 14 and *P* = 0.0096, *t* = 2.572, df = 18; Figure 4(e)). There was a tendency to longer latency of immobility during these measurements; a significant increase was found on Day 2 of testing of mice treated with 750 mcg/kg (*P* = 0.0169, *t* = 2.352, df = 14). Animals that received T2 at the dose 750 mcg/kg of T2 after the first test session showed a tendency to decreased immobility (*P* = 0.0734, *t* = 1.536, and df = 14) and significantly increased latency of immobility (*P* = 0.006, *t* = 2.840, df = 14) on Day 2; experimental groups have displayed no behavioural differences prior to the treatment, on Day 1 (Figure 4(c)).

**Figure 5:**
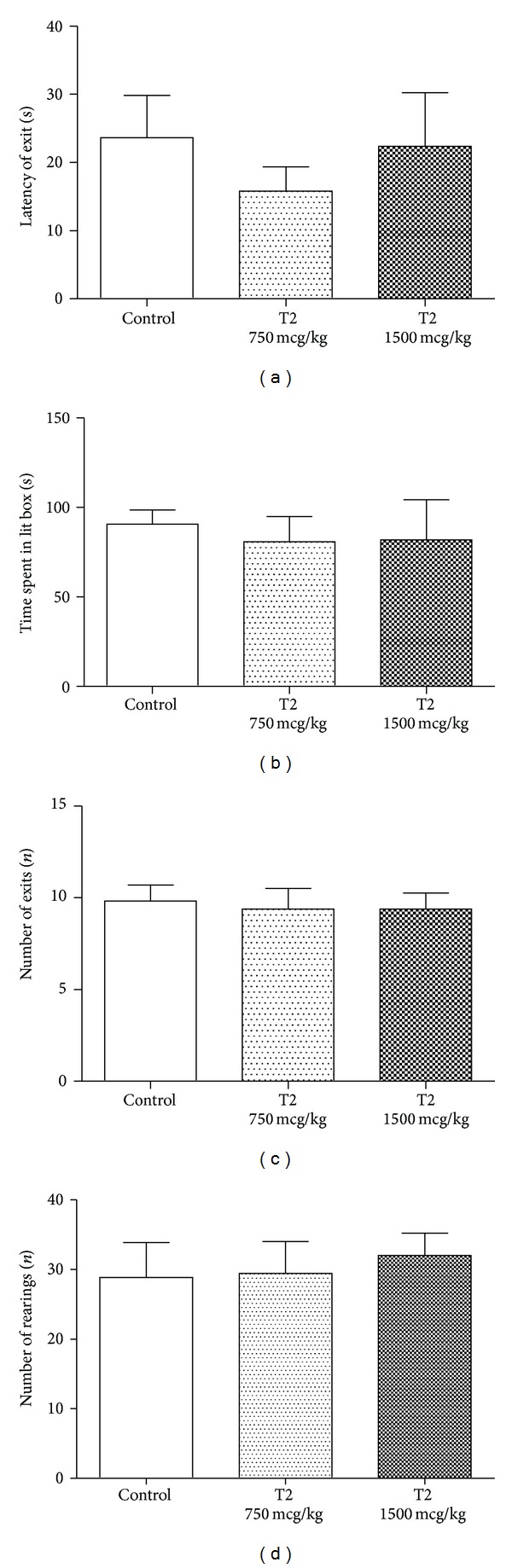
3,5-Diiodo-L-thyronine T2-treated mice display unaltered anxiety-like and locomotor behaviour. In the dark-light box, mice that were treated with T2 at the dose 750 mcg/kg or 1500 mcg/kg did not differ from vehicle-treated mice in (a) the time spent in the lit compartment, (b) latency of exit in the lit area, and (c) number of exits to the lit box. (d) In the novel cage, mice that received T2 at the dose 750 mcg/kg or 1500 mcg/kg did not differ from vehicle-treated mice in number of rearings. All groups constituted 9 mice.

Dosing with 750 or 1500 mcg/kg did not alter anxiety-like behaviour of C57Bl6J mice (*data not shown*) and CD1 mice, as shown by unaffected latencies of the first exit to the lit compartment (one-way ANOVA, *F* = 0.4640, *R* ^2^ = 0.0662, and *P* = 0.6388; Figure 5(a)), time spent therein (one-way ANOVA, *F* = 2.390, *R* ^2^ = 0.2688, and *P* = 0.1307; Figure 5(b)), and number of exits (one-way ANOVA, *F* = 0.07238, *R* ^2^ = 0.01101, and *P* = 0.9305; Figure 5(c)). The number of rearings was unaltered (one-way ANOVA, *F* = 0.1395, *R* ^2^ = 0.02101, and *P* = 0.8711; Figure 5(d)).

## 4. Discussion

The present study highlights hippocampal gene expression of DIO2 and DIO3, enzymes involved in the thyroid hormone regulation, as a molecular correlate of antidepressant-like effects in a stress-induced depressive syndrome in mice. The present study also identifies increased hippocampal gene expression of DIO2 alone in the stress resilient cohort of mice. While these changes in enzyme expression levels may not necessary generate elevated levels of central thyroid hormone, the ability of administrated T2 to reverse parameters of behavioural despair supports the hypothesis for T2 as a contributor to antidepressant activity and suggests that the administration or augmentation of T2 production may be of value in the clinic.

Stress-induced anhedonia in mice [34, 35, 40] resulting in decreased intake or preference for palatable solutions (such as a sucrose solution) is interpreted as a decreased ability to experience pleasure, a core feature of human depression [41]. The stressed mice treated with imipramine in this study retained a sucrose preference, which is in agreement with reports from our lab and others, that conventional antidepressant treatments are able to prevent the stress-induced decrease in sucrose preference [29, 42, 43]. In addition to the effects on sucrose preference, we showed that treatment with imipramine had antidepressant-like effects in the tail suspension test, and coat state was also improved by imipramine. Coat disintegration in rodents is due to reduced motivation for self-cleaning and is used as an important feature of a depressive-like state in preclinical models [44]. Improvements in coat state have accordingly been documented for antidepressant treatments in rodent models of depression [36, 40, 42]. Imipramine diminished stress-induced loss of body weight. It has been previously shown that antidepressant treatment results in a restoration of body weight [40, 42, 43]. Thus it can easily be argued that imipramine, at the dose employed, has been effective as an antidepressant as a consequence of the improvement observed in all of these outcomes. This is of note because we used a much lower dose than has been used by convention. The use of a lower dose was indicated after we showed that higher doses of imipramine are associated with significant side effects in naive animals [39]. Such side effects might have been expected to confound our experimental outcomes, and thus it was important to be able to validate this lower dose.

Illumina microarray data and our RTPCR assays suggested elevated hippocampal gene expression of DIO2, encoding a key enzyme of the synthesis of T2 and T3 hormones, in stressed mice that received imipramine and in stressed mice that are resilient versus mice susceptible to depressive syndrome. RTPCR also showed a significant increase in DIO3 expression in stressed imipramine-treated mice, but no other group differences were significant. As DIO2 is an enzyme of thyroid hormone synthesis in the brain, DIO3 inactivates thyroid hormones, and simultaneous activation of both biosynthesis and degradation of neurochemically active factors often accompanies their enhanced turnover. In line with our findings, subchronic antidepressant and antipsychotic treatments in a rat induced more pronounced brain changes of DIO2 than of DIO3, which have occurred in the same direction [15]. In support of our results, exposure of a rat to 24 h sleep deprivation, commonly used as nonpharmacological antidepressant treatment, has increased the activities of DIO2 in the hippocampus and several more brain structures, without affecting DIO3; of note, no such changes were found in the amygdala in these experiments [15]. This study also has shown that desipramine, an antidepressant that is similar to the one used here imipramine, has induced dose-dependent increases of DIO2 in the cortex, whereas no changes in the DIO3 were observed [15]. Notably, among deiodinases 1, 2, and 3, DIO2 in particular was found to be the highly sensitive to many different kinds of influence that are believed to be capable of inducing the changes in thyroid hormone concentrations in the CNS and neuronal activity [15]. Other studies showed that mice lacking DIO2 were demonstrated to have deficits in the hippocampus-dependent learning [45], obesity with glucose intolerance [46] that is a well-established indicator of depressive disorder. Since DIO2 gene expression was increased in our study in resilient (nonanhedonic) mice or animals that received imipramine, it can be speculated that elevated synthesis of thyroid hormones in the hippocampus is associated with resilience to a depressive-like syndrome. To the best of our knowledge, no such changes in the DIO2 have been reported until now in relation to a susceptibility to experimentally induced depressive-like status in animal models of depression.

Our molecular biology results are indicative of altered central thyroid hormone concentrations, but they are not conclusive, and direct measurements of T2 and T3 in the brain are required. However, the RTPCR results prompted us to investigate the effect of a bolus injection on the immobilization behaviour in the tail suspension test in C57BL6J and CD1 mice. All the mice treated with T2 prior to the first test session displayed shortened time spent immobile on both days of testing. Our results, obtained in two treatment protocols on two mouse strains, suggest an antidepressant-like effect of used here dosing with T2. Present data with T2 are in accordance with our previous observations that have shown more pronounced antidepressant effects of acutely injected SSRIs and tricyclics, which are administrated prior to the first session of the tail suspension experiment, than afterwards, both in the C57BL6J and CD1 mice (Strekalova, *unpublished results*). In most of the cases, the antidepressant-like action of bolus dosing with conventional antidepressants is often detectable in mice only on the second testing session. Here, however, a reduction of a depressive-like behaviour in T2-treated animals was observed on both Day 1 and Day 2 of the experiment.

Next, we found that both C57BL/6J and CD1 mice showed no changes in the parameters of anxiety and locomotion when treated with T2 at the doses that have elicited a decrease in the immobility time. Namely, in a classical mouse paradigm for anxiety-like behaviour [29, 34], the dark-light box, CD1 mice that were treated with T2 at the dose 750 mcg/kg or 1500 mcg/kg did not show any signs of altered parameters of anxiety-like behaviour. In the novel cage, a sensitive paradigm for minor changes in mouse activity [38, 39], CD1 mice that received T2 at the dose 750 mcg/kg or 1500 mcg/kg did not display any locomotor alternations; similar results in both tests were obtained in C57BL/6J (*data not shown*). This rules out potential confounds in the evaluation of the antidepressant-like activity of the treatment with T2 that might interfere with the changes in emotionality and general locomotor activity.

The antidepressant-like effects of T2 in the tail suspension paradigm suggest specific brain mechanisms of transport of peripheral T2. Among several thyroid hormone transporters, recent studies have identified monocarboxylate transporter 8 (MCT8) molecule as a very active and specific transporter of all thyroid hormones, T4, T3, and T2, which transports them with comparable uptake rates at various locations [47] and is highly expressed in hippocampus, olfactory bulb, cerebral cortex, amygdale, and choroid plexus [48]. A hypothesis was derived for the mechanism of the transport of thyroid hormones from the bloodstream to the brain involving thyroid hormone transporter synthesized in choroid plexus and secreted into the cerebrospinal fluid [49]. While several mechanisms of action of T2 can be proposed, including above-discussed effects of this hormone on monoamine and integrin receptors, PI3 K-Akt signaling and TRH, given accumulated evidences concerning eminent effects of T2 on the mitochondrial respiratory chain [20, 21], biogenesis [17], and calcium and NO signalling [22], they are likely to be due to the enhancement of mitochondrial functions, a validated target of antidepressant therapy [19].

In conclusion, our data further suggest an importance of central thyroid hormone signalling in depressive-like changes that can be targeted to improve available therapy of this syndrome. Our data also provide support for the translation of T2 enhancing therapy, perhaps in conjunction with conventional therapy, especially when a rapid therapeutic effect is relevant and/or metabolic parameters of the patients are compromised.
